# Editorial: Sequencing technologies in advancing veterinary and zoonotic infection research

**DOI:** 10.3389/fcimb.2026.1950388

**Published:** 2026-08-25

**Authors:** Henry M. Kariithi, Valerie J. Carabetta, Christina L. Leyson, Iryna V. Goraichuk

**Affiliations:** 1 Biotechnology Research Institute, Kenya Agricultural and Livestock Research Organization, Nairobi, Kenya; 2 Cooper Medical School of Rowan University, Camden, NJ, United States; 3 College of Veterinary Medicine, Auburn University, Auburn, AL, United States; 4 ASRT, Inc., Smyrna, GA, United States

**Keywords:** antimicrobial resistance, genomic surveillance, host adaptation, metagenomics, molecular epidemiology, outbreak investigation, pathogen genomics

## 1 Sequencing as a transformative framework

Infectious disease threats are wide-ranging, global and interconnected, affecting livestock productivity and revenue, food security, wildlife conservation, and human health. Disease emergence and transmission are driven by ecological, environmental and anthropogenic factors, including animal movement and trade, agricultural intensification, inadequate biosecurity, climate change, and wildlife–livestock–human interfaces.

Integration of genomic sequencing with epidemiological surveillance and appropriate metadata generates high-resolution, actionable information. Depending on the approach and analytical objective, outputs may include genome recovery and genotyping of causative agents, characterization of mixed and coinfections, identification of mutations, reassortment and recombination events, discovery of novel pathogens, and profiling of virulence determinants (Gardy and Loman, 2018; Armstrong et al., 2019). Sequencing approaches span a continuum suited to different questions, from targeted, high-resolution characterization of known pathogens to broader, pathogen-agnostic discovery (Rong et al., 2026).

The 16 papers assembled in this Research Topic collectively illustrate complementary roles of sequencing technologies across this continuum, from methodological optimization and diagnostic investigations to genomic surveillance, evolutionary analyses, and biological interpretation. Together, these studies demonstrate the expanding role of sequencing technologies in advancing veterinary and zoonotic infection research.

## 2 Making sequencing faster, more accessible, and fit for purpose

The value of genomic sequencing depends on the entire workflow rather than the sequencing instrument alone. Because no workflow is universally optimal, methods should be selected according to the biological question or application, sample type and quality, expected pathogen abundance, genome characteristics, required throughput and turnaround time, and available computational resources (Berry et al., 2020).

Chaves et al. highlighted the growing role of Oxford Nanopore Technologies (ONT) in veterinary medicine, emphasizing its portability, real-time data generation, long-read capability, relatively low instrumentation costs, and suitability for decentralized or field-based sequencing. As with any sequencing technology, optimal performance depends on appropriate sample preparation, quality control, bioinformatics, and trained personnel. Sequencing platform selection should therefore be guided by the intended application. Accordingly, short-read technologies remain preferable for high per-base accuracy, deep multiplexing, and sensitive detection of low-frequency variants, whereas ONT offers unique advantages for long-read sequencing, rapid turnaround, and deployment in resource-limited or field settings.

Workflow optimization is equally important. Goraichuk et al. demonstrated that incremental improvements in RT-PCR chemistry, primer design, purification methods, and sample automation substantially improved genome coverage and sequencing performance for influenza A virus. Building on this work, Goraichuk and Suarez further demonstrated that custom barcoded primers for ONT substantially reduced library preparation time while maintaining sequencing quality, thereby improving turnaround for influenza genomic surveillance. Together, these complementary studies underscore that genome completeness and operational efficiency depend as much on workflow optimization as on the sequencing platform itself.

Target enrichment represents another important strategy for improving sequencing efficiency. Using avian metapneumovirus as a model, Álvarez-Narváez et al. showed that tiled amplicon-based sequencing substantially improved genome recovery from samples with high host background while remaining compatible with both short- and long-read platforms. Because this approach depends on prior sequence knowledge, continued surveillance and periodic primer updates are essential as viral populations continue to evolve.

Finally, Kattoor et al. demonstrated that multiplex targeted sequencing can simultaneously detect multiple pathogens associated with canine neurological and reproductive diseases while providing strain-level information. Such syndromic approaches bridge the gap between conventional single-target PCR and untargeted metagenomics, although expanding the number of targets may reduce analytical sensitivity and increase assay complexity.

## 3 From unexplained disease to pathogen discovery and outbreak resolution

Pathogen-agnostic sequencing approaches complement hypothesis-driven diagnostic methods in investigations of unresolved, atypical, multifactorial or multi-agent infectious diseases. Untargeted metagenomic sequencing is hypothesis-free in target selection, but not interpretation-free, meaning that biological and clinical judgement are essential for distinguishing true pathogens from commensals, incidental detections, and background contaminants (Miller and Chiu, 2022).

The untargeted metagenomic study by Kariithi et al. demonstrated the complexity of disease-associated viral communities through co-detection and genomic characterization of multiple genetically diverse enteric viruses in pooled poultry samples, revealing distinct genomic constellations and reassortment-related patterns. The study illustrates that metagenomic sequencing can comprehensively define complex viral communities. However, determining the relative contribution of individual pathogens to disease and establishing causality still require integration with clinical, pathological, and epidemiological evidence. Such approaches provide a more complete etiological picture and help prioritize candidate pathogens for subsequent investigations.

Beyond pathogen detection alone, genomic sequences place outbreak-associated pathogens within their evolutionary context, enabling comparison with prevailing strains and strengthening epidemiological investigations (Robinson et al., 2013). Sabyrzhan et al. demonstrated how sequencing complemented conventional diagnostics during investigation of a fatal canine gastroenteritis outbreak by confirming viral coinfection and providing genomic insights into the detected pathogens.

Zoonotic diagnosis depends on the combination of clinical signs, exposure history, and laboratory evidence; atypical zoonotic infections are difficult to diagnose via conventional methods. Song et al. illustrated this through an atypical presentation of cat-scratch disease in which sequencing contributed to establishing an otherwise difficult diagnosis.

Metatranscriptomics further expands sequencing beyond pathogen detection to the integrated characterization of active microbial communities and host responses (Destras et al., 2026). Li et al. extended this approach to viral ecology by characterizing the horse gut RNA virome and identifying shared viral networks among horses, humans, and other domestic animals.

## 4 Whole-genome sequencing for surveillance, evolution, and transmission

In addition to identifying the causative agent, WGS can resolve genetic lineages, spatial and temporal clustering, reassortment and recombination events, transmission pathways, introduction into and persistence within local populations, and outbreak boundaries. Collectively, these capabilities provide the genomic context needed for surveillance and disease control decisions (Struelens et al., 2024).

Because migratory birds, particularly waterfowl, are natural reservoirs of avian influenza viruses (Olsen et al., 2006), surveillance of wild birds provides an early warning system for detecting viruses with potential to be introduced into poultry and, in some cases, human populations. The report by Si et al. on H5N1 and H5N9 highly pathogenic avian influenza viruses demonstrated the value of sequencing at the beginning of migration by enabling early recognition of newly introduced lineages. It further identified complete gene constellations, reassortment patterns and mutations with potential implications for host range and pathogenicity, thereby informing surveillance, diagnostic, and risk assessment efforts.

Even recovery of a single isolate can substantially expand genomic knowledge, particularly for rare or poorly characterized pathogens. The incidental detection and genomic characterization of the first coding-complete Avian orthoavulavirus 16 (AOAV-16) genome from a cinereous vulture by Son et al. during influenza surveillance extended the known host range beyond wild *Anseriformes*, the avian order in which the virus has predominantly been detected. Considering the scarcity of AOAV-16 genomic resources, such reports expand reference databases, which are critical for whole-genome-based classifications, and improve understanding of the host range and geographical distribution of AOAV-16.

Beyond expanding genomic resources for rare viruses, WGS also strengthens genomic epidemiology of zoonotic bacterial pathogens. This is illustrated by Yang et al., who characterized the first reported human isolate of *Brucella canis* using comparative genomics and whole-genome SNP analysis, thereby clarifying its phylogenetic relationships and highlighting the need for enhanced surveillance. Similarly, Yang et al. used integrated genomic analyses of *B. abortus* isolates from humans and cattle to define their population structure, phylogenetic relationships, and regional transmission patterns, illustrating how WGS strengthens surveillance and source attribution of important zoonotic bacteria.

## 5 Genomics and transcriptomics in pathogen biology

Beyond identifying and tracking pathogens, genomic sequencing can provide important insights into biological characteristics and host-pathogen interactions. However, genomic datasets predict biological potential rather than expressed phenotype or disease outcome. Their interpretation therefore requires integration with complementary laboratory, pathological, and epidemiological evidence (Balloux et al., 2018; Sheppard et al., 2018).

In their study of avian pathogenic *Escherichia coli* O145, Tan et al. combined WGS, experimental infection, pathology, and *in vivo* transcriptomics to investigate pathogenicity and host adaptation. Transcriptomic comparison of bacteria colonizing duck liver with those grown *in vitro* identified 87 differentially expressed genes, including genes associated with metabolism and host adaptation, as well as changes in central metabolic pathways and regulatory small RNAs. The study illustrates how genomic potential can be linked to genes and pathways expressed under specific host conditions, although the biological functions of many identified transcripts remain to be experimentally validated.

Wang et al. further demonstrated that multidimensional characterization of newly detected lineages benefits from the integration of genomic and phenotypic analyses. Hybrid WGS defined the phylogenetic placement, resistome, virulome, and mobile genetic elements of the novel *Acinetobacter baumannii* ST3475 lineage, whereas cell-based assays and animal models demonstrated enhanced biofilm formation, adhesion, invasion and pathogenicity. Although phylogenetic clustering suggested possible adaptation to the bovine mammary niche, broader surveillance and epidemiological investigations are needed to determine its zoonotic potential.

Finally, Mohamed et al. synthesized evidence on virulence-associated genes in *Campylobacter jejuni* and *C. coli* from food and human sources across Gulf Cooperation Council countries. The review highlighted heterogeneous virulence profiles and important regional evidence gaps, particularly the limited number of human studies and uncertainty surrounding the relationship between gene carriage and pathogenicity. Such regional syntheses can support the development of harmonized surveillance strategies and help prioritize virulence determinants for functional investigation.

## 6 Cross-cutting lessons and future directions

The studies published in this Research Topic collectively demonstrate how sequencing has evolved beyond pathogen detection into a versatile platform supporting diagnostics, pathogen discovery, outbreak investigation, epidemiological surveillance, comparative genomics, and functional investigations. Across diverse pathogens and host species, sequencing consistently provided greater resolution than conventional diagnostic approaches while expanding understanding of pathogen diversity, evolution, and biology.

An equally important lesson emerging from this collection is that genomic data are most valuable when interpreted alongside complementary clinical, pathological, phenotypic and epidemiological evidence. Likewise, the methodological studies demonstrate that successful implementation depends not only on sequencing technologies but also on optimized workflows tailored to specific diagnostic, surveillance, and research applications. These findings underscore the importance of matching sequencing strategies, analytical methods, and validation approaches to the intended application.

Taken together, these studies reflect the continuing transition of sequencing from a specialized research tool to an increasingly accessible platform supporting veterinary and zoonotic infectious disease investigations. Continued progress will require optimized, standardized workflows, integration of genomic data with complementary biological and epidemiological evidence, sustained genomic surveillance, and multidisciplinary collaboration within a One Health framework. These priorities will be essential for strengthening infectious disease preparedness, detection and response to both existing and emerging disease threats.
